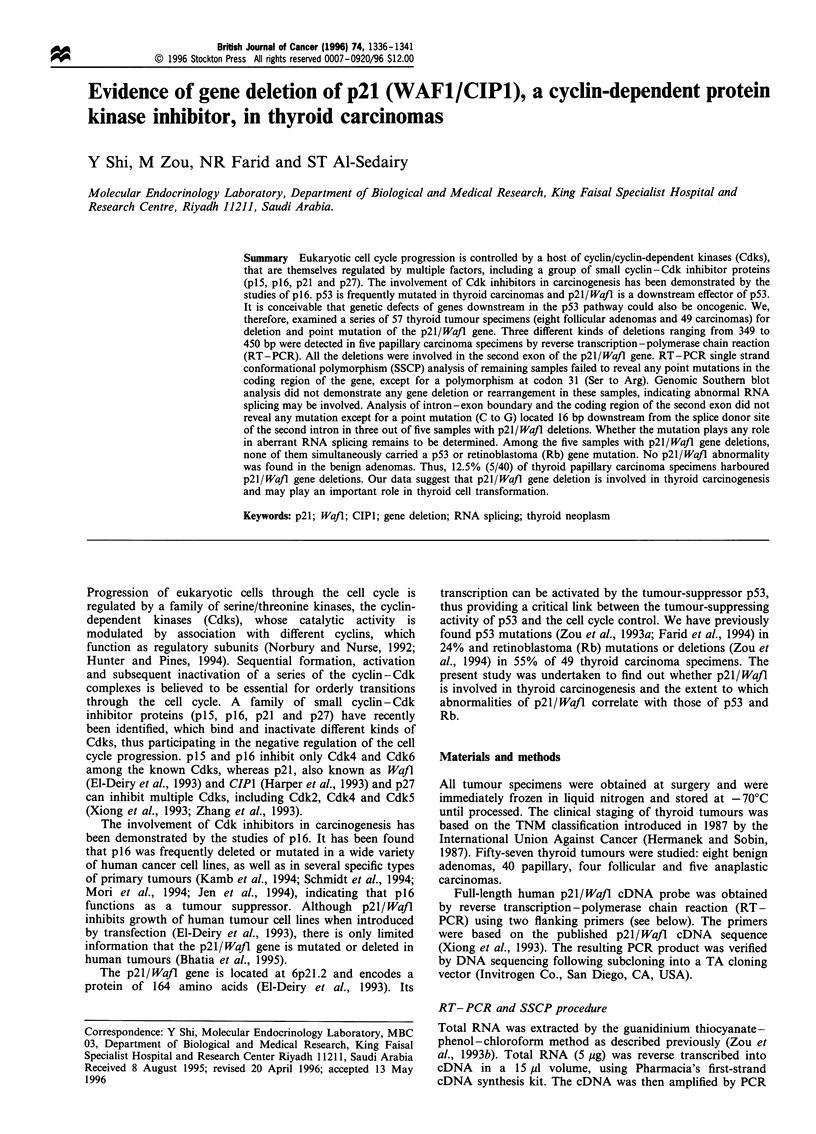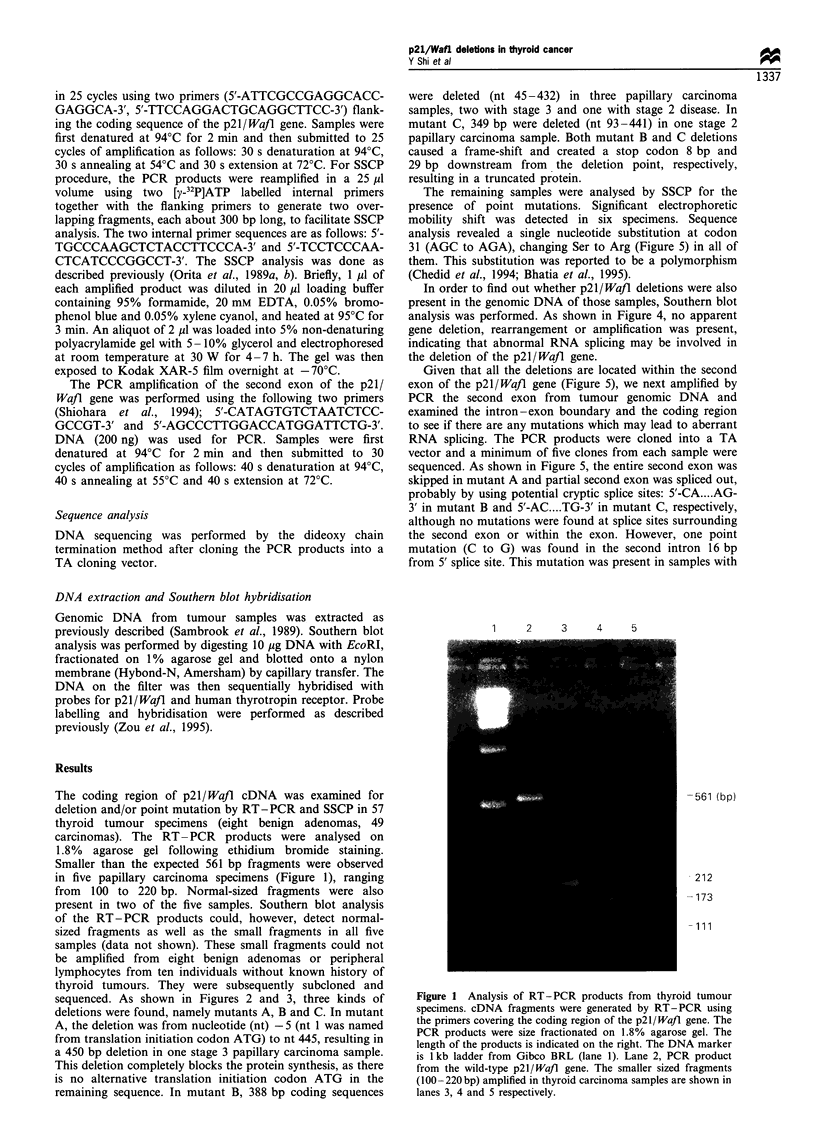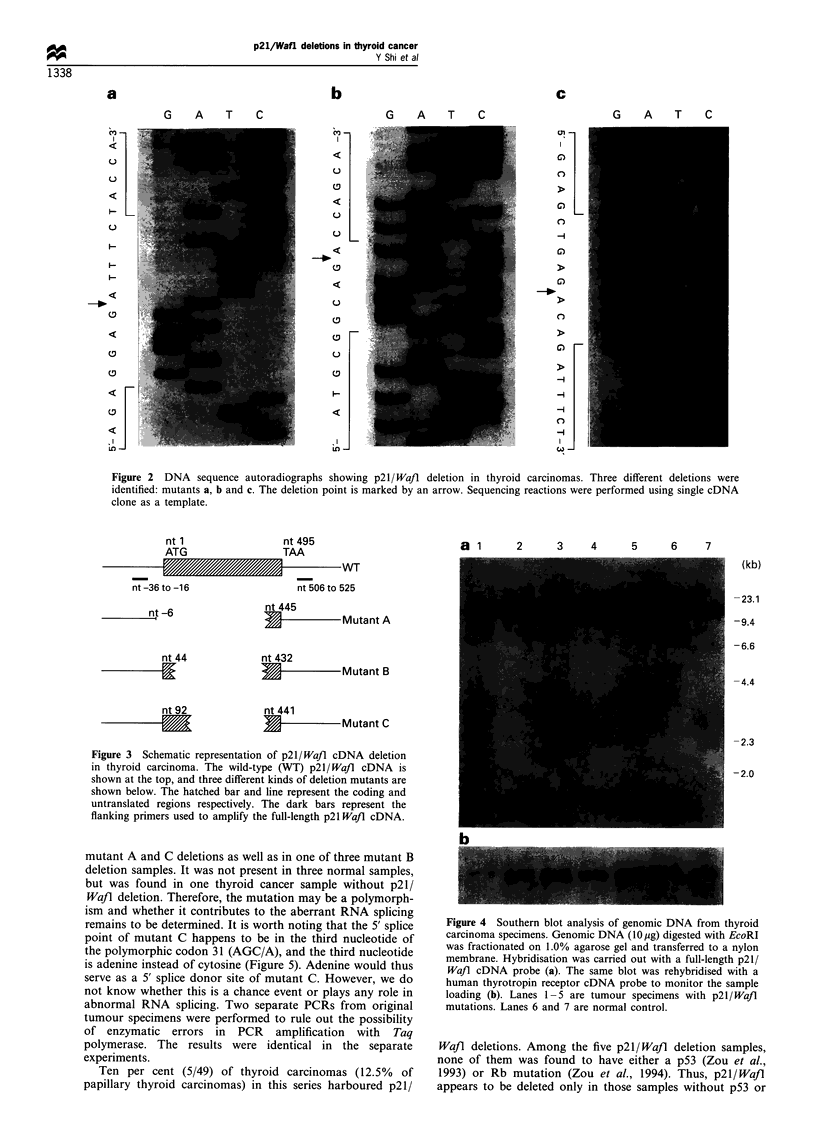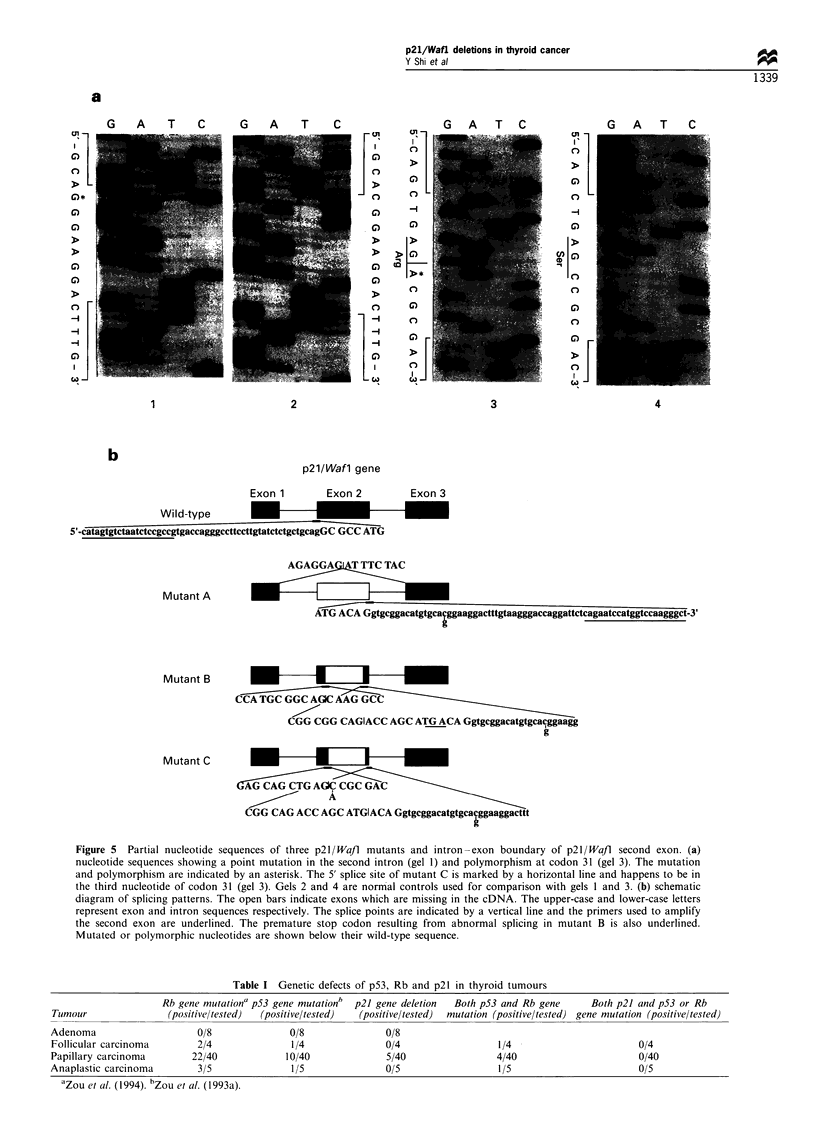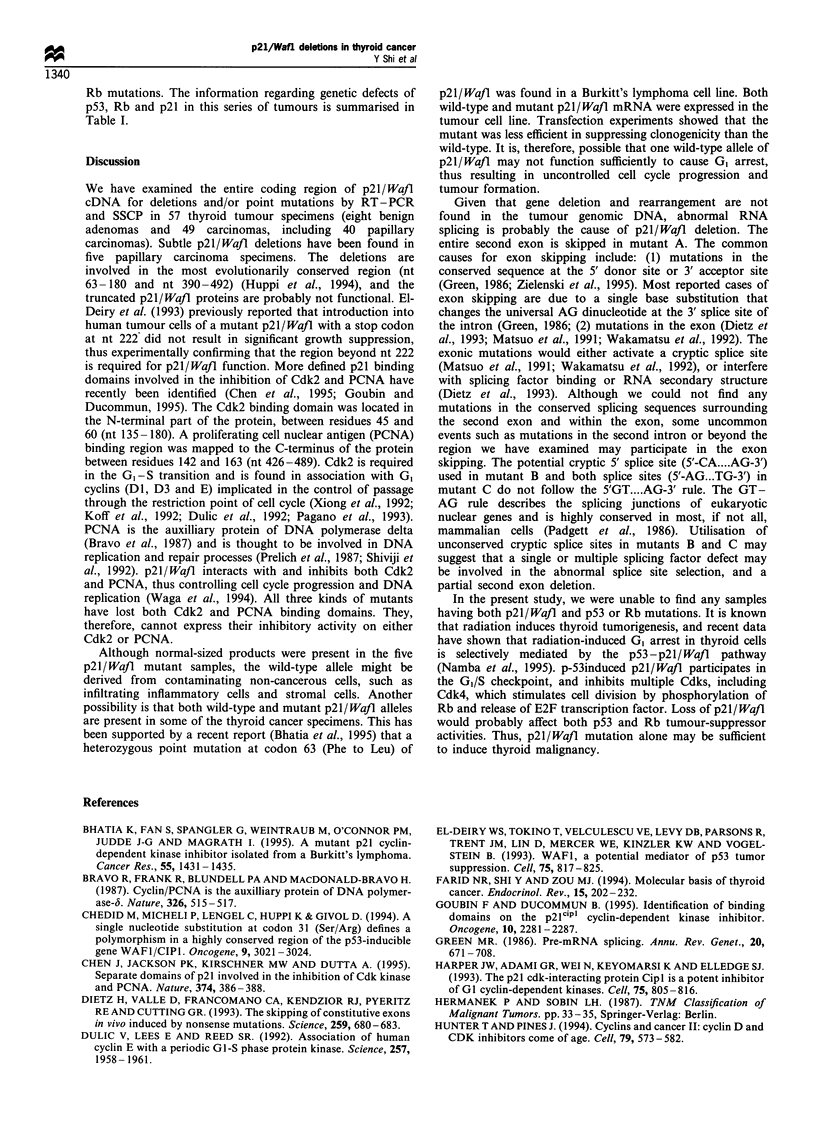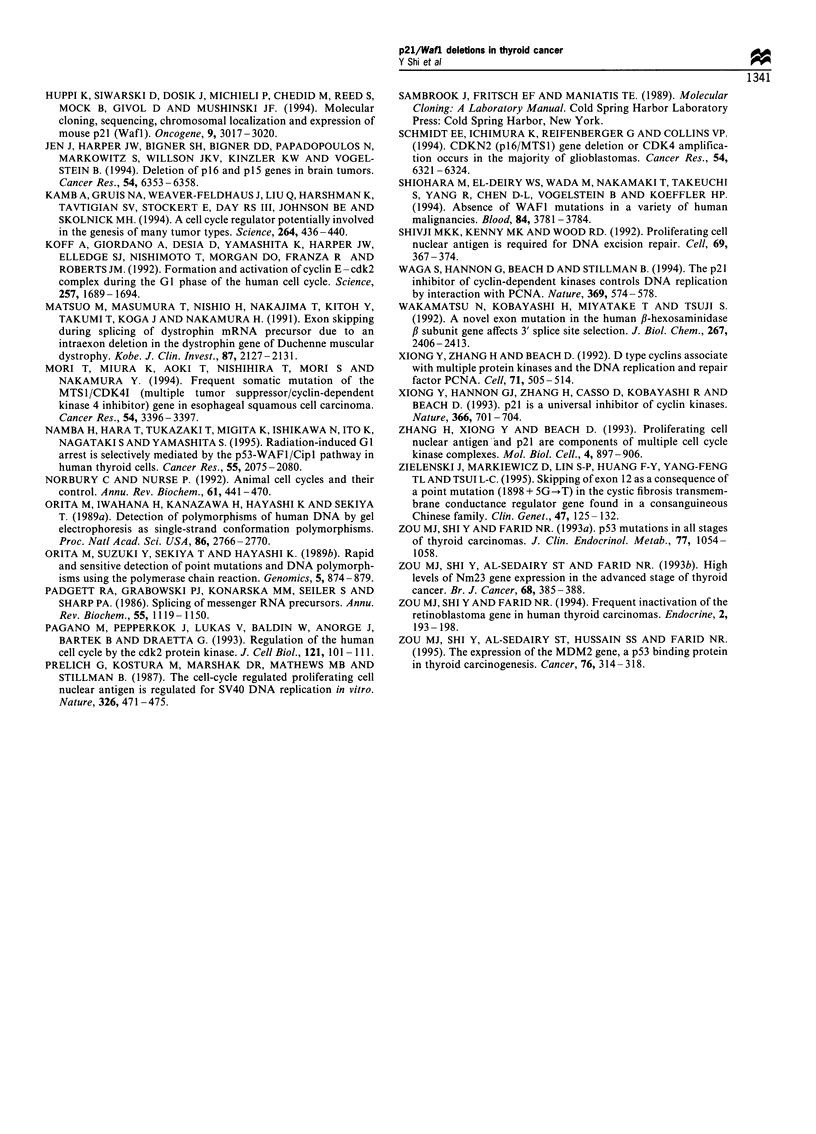# Evidence of gene deletion of p21 (WAF1/CIP1), a cyclin-dependent protein kinase inhibitor, in thyroid carcinomas.

**DOI:** 10.1038/bjc.1996.546

**Published:** 1996-11

**Authors:** Y. Shi, M. Zou, N. R. Farid, S. T. al-Sedairy

**Affiliations:** Department of Biological and Medical Research, King Faisal Specialist Hospital and Research Centre, Riyadh, Saudi Arabia.

## Abstract

**Images:**


British Journal of Cancer (1996) 74, 1336-1341
© 1996 Stockton Press All rights reserved 0007-0920/96 $12.00

### Evidence of gene deletion of p21 (WAF1/CIP1), a cyclin-dependent protein kinase inhibitor, in thyroid carcinomas

Y Shi, M Zou, NR Farid and ST Al-Sedairy

*Molecular Endocrinology Laboratory, Department of Biological and Medical Research, King Faisal Specialist Hospital and Research Centre, Riyadh 11211, Saudi Arabia.*

**Summary** Eukaryotic cell cycle progression is controlled by a host of cyclin/cyclin-dependent kinases (Cdks), that are themselves regulated by multiple factors, including a group of small cyclin–Cdk inhibitor proteins (p15, p16, p21 and p27). The involvement of Cdk inhibitors in carcinogenesis has been demonstrated by the studies of p16. p53 is frequently mutated in thyroid carcinomas and p21/*Waf1* is a downstream effector of p53. It is conceivable that genetic defects of genes downstream in the p53 pathway could also be oncogenic. We, therefore, examined a series of 57 thyroid tumour specimens (eight follicular adenomas and 49 carcinomas) for deletion and point mutation of the p21/*Waf1* gene. Three different kinds of deletions ranging from 349 to 450 bp were detected in five papillary carcinoma specimens by reverse transcription–polymerase chain reaction (RT–PCR). All the deletions were involved in the second exon of the p21/*Waf1* gene. RT–PCR single strand conformational polymorphism (SSCP) analysis of remaining samples failed to reveal any point mutations in the coding region of the gene, except for a polymorphism at codon 31 (Ser to Arg). Genomic Southern blot analysis did not demonstrate any gene deletion or rearrangement in these samples, indicating abnormal RNA splicing may be involved. Analysis of intron–exon boundary and the coding region of the second exon did not reveal any mutation except for a point mutation (C to G) located 16 bp downstream from the splice donor site of the second intron in three out of five samples with p21/*Waf1* deletions. Whether the mutation plays any role in aberrant RNA splicing remains to be determined. Among the five samples with p21/*Waf1* gene deletions, none of them simultaneously carried a p53 or retinoblastoma (Rb) gene mutation. No p21/*Waf1* abnormality was found in the benign adenomas. Thus, 12.5% (5/40) of thyroid papillary carcinoma specimens harboured p21/*Waf1* gene deletions. Our data suggest that p21/*Waf1* gene deletion is involved in thyroid carcinogenesis and may play an important role in thyroid cell transformation.

**Keywords:** p21; *Waf1*; CIP1; gene deletion; RNA splicing; thyroid neoplasm

Progression of eukaryotic cells through the cell cycle is regulated by a family of serine/threonine kinases, the cyclin-dependent kinases (Cdks), whose catalytic activity is modulated by association with different cyclins, which function as regulatory subunits (Norbury and Nurse, 1992; Hunter and Pines, 1994). Sequential formation, activation and subsequent inactivation of a series of the cyclin–Cdk complexes is believed to be essential for orderly transitions through the cell cycle. A family of small cyclin–Cdk inhibitor proteins (p15, p16, p21 and p27) have recently been identified, which bind and inactivate different kinds of Cdks, thus participating in the negative regulation of the cell cycle progression. p15 and p16 inhibit only Cdk4 and Cdk6 among the known Cdks, whereas p21, also known as *Waf1* (El-Deiry *et al.*, 1993) and *CIP1* (Harper *et al.*, 1993) and p27 can inhibit multiple Cdks, including Cdk2, Cdk4 and Cdk5 (Xiong *et al.*, 1993; Zhang *et al.*, 1993).

The involvement of Cdk inhibitors in carcinogenesis has been demonstrated by the studies of p16. It has been found that p16 was frequently deleted or mutated in a wide variety of human cancer cell lines, as well as in several specific types of primary tumours (Kamb *et al.*, 1994; Schmidt *et al.*, 1994; Mori *et al.*, 1994; Jen *et al.*, 1994), indicating that p16 functions as a tumour suppressor. Although p21/*Waf1* inhibits growth of human tumour cell lines when introduced by transfection (El-Deiry *et al.*, 1993), there is only limited information that the p21/*Waf1* gene is mutated or deleted in human tumours (Bhatia *et al.*, 1995).

The p21/*Waf1* gene is located at 6p21.2 and encodes a protein of 164 amino acids (El-Deiry *et al.*, 1993). Its transcription can be activated by the tumour-suppressor p53, thus providing a critical link between the tumour-suppressing activity of p53 and the cell cycle control. We have previously found p53 mutations (Zou *et al.*, 1993*a*; Farid *et al.*, 1994) in 24% and retinoblastoma (Rb) mutations or deletions (Zou *et al.*, 1994) in 55% of 49 thyroid carcinoma specimens. The present study was undertaken to find out whether p21/*Waf1* is involved in thyroid carcinogenesis and the extent to which abnormalities of p21/*Waf1* correlate with those of p53 and Rb.

### Materials and methods

All tumour specimens were obtained at surgery and were immediately frozen in liquid nitrogen and stored at −70°C until processed. The clinical staging of thyroid tumours was based on the TNM classification introduced in 1987 by the International Union Against Cancer (Hermanek and Sobin, 1987). Fifty-seven thyroid tumours were studied: eight benign adenomas, 40 papillary, four follicular and five anaplastic carcinomas.

Full-length human p21/*Waf1* cDNA probe was obtained by reverse transcription–polymerase chain reaction (RT–PCR) using two flanking primers (see below). The primers were based on the published p21/*Waf1* cDNA sequence (Xiong *et al.*, 1993). The resulting PCR product was verified by DNA sequencing following subcloning into a TA cloning vector (Invitrogen Co., San Diego, CA, USA).

### *RT–PCR and SSCP procedure*

Total RNA was extracted by the guanidinium thiocyanate–phenol–chloroform method as described previously (Zou *et al.*, 1993*b*). Total RNA (5 μg) was reverse transcribed into cDNA in a 15 μl volume, using Pharmacia's first-strand cDNA synthesis kit. The cDNA was then amplified by PCR

Correspondence: Y Shi, Molecular Endocrinology Laboratory, MBC 03, Department of Biological and Medical Research, King Faisal Specialist Hospital and Research Center Riyadh 11211, Saudi Arabia
Received 8 August 1995; revised 20 April 1996; accepted 13 May 1996

in 25 cycles using two primers (5′-ATTCGCCGAGGCACC-GAGGCA-3′, 5′-TTCCAGGACTGCAGGCTTCC-3′) flanking the coding sequence of the p21/*Waf1* gene. Samples were first denatured at 94°C for 2 min and then submitted to 25 cycles of amplification as follows: 30 s denaturation at 94°C, 30 s annealing at 54°C and 30 s extension at 72°C. For SSCP procedure, the PCR products were reamplified in a 25 $\mu$l volume using two [$\gamma$-$^{32}$P]ATP labelled internal primers together with the flanking primers to generate two overlapping fragments, each about 300 bp long, to facilitate SSCP analysis. The two internal primer sequences are as follows: 5′-TGCCCAAGCTCTACCTTCCCA-3′ and 5′-TCCTCCCAA-CTCATCCCGGCCT-3′. The SSCP analysis was done as described previously (Orita *et al*., 1989*a*, *b*). Briefly, 1 $\mu$l of each amplified product was diluted in 20 $\mu$l loading buffer containing 95% formamide, 20 mM EDTA, 0.05% bromophenol blue and 0.05% xylene cyanol, and heated at 95°C for 3 min. An aliquot of 2 $\mu$l was loaded into 5% non-denaturing polyacrylamide gel with 5–10% glycerol and electrophoresed at room temperature at 30 W for 4–7 h. The gel was then exposed to Kodak XAR-5 film overnight at −70°C.

The PCR amplification of the second exon of the p21/*Waf1* gene was performed using the following two primers (Shiohara *et al*., 1994); 5′-CATAGTGTCTAATCTCC-GCCGT-3′ and 5′-AGCCCTTGGACCATGGATTCTG-3′. DNA (200 ng) was used for PCR. Samples were first denatured at 94°C for 2 min and then submitted to 30 cycles of amplification as follows: 40 s denaturation at 94°C, 40 s annealing at 55°C and 40 s extension at 72°C.

*Sequence analysis*

DNA sequencing was performed by the dideoxy chain termination method after cloning the PCR products into a TA cloning vector.

*DNA extraction and Southern blot hybridisation*

Genomic DNA from tumour samples was extracted as previously described (Sambrook *et al*., 1989). Southern blot analysis was performed by digesting 10 $\mu$g DNA with *Eco*RI, fractionated on 1% agarose gel and blotted onto a nylon membrane (Hybond-N, Amersham) by capillary transfer. The DNA on the filter was then sequentially hybridised with probes for p21/*Waf1* and human thyrotropin receptor. Probe labelling and hybridisation were performed as described previously (Zou *et al*., 1995).

### Results

The coding region of p21/*Waf1* cDNA was examined for deletion and/or point mutation by RT–PCR and SSCP in 57 thyroid tumour specimens (eight benign adenomas, 49 carcinomas). The RT–PCR products were analysed on 1.8% agarose gel following ethidium bromide staining. Smaller than the expected 561 bp fragments were observed in five papillary carcinoma specimens (Figure 1), ranging from 100 to 220 bp. Normal-sized fragments were also present in two of the five samples. Southern blot analysis of the RT–PCR products could, however, detect normal-sized fragments as well as the small fragments in all five samples (data not shown). These small fragments could not be amplified from eight benign adenomas or peripheral lymphocytes from ten individuals without known history of thyroid tumours. They were subsequently subcloned and sequenced. As shown in Figures 2 and 3, three kinds of deletions were found, namely mutants A, B and C. In mutant A, the deletion was from nucleotide (nt) −5 (nt 1 was named from translation initiation codon ATG) to nt 445, resulting in a 450 bp deletion in one stage 3 papillary carcinoma sample. This deletion completely blocks the protein synthesis, as there is no alternative translation initiation codon ATG in the remaining sequence. In mutant B, 388 bp coding sequences were deleted (nt 45–432) in three papillary carcinoma samples, two with stage 3 and one with stage 2 disease. In mutant C, 349 bp were deleted (nt 93–441) in one stage 2 papillary carcinoma sample. Both mutant B and C deletions caused a frame-shift and created a stop codon 8 bp and 29 bp downstream from the deletion point, respectively, resulting in a truncated protein.

The remaining samples were analysed by SSCP for the presence of point mutations. Significant electrophoretic mobility shift was detected in six specimens. Sequence analysis revealed a single nucleotide substitution at codon 31 (AGC to AGA), changing Ser to Arg (Figure 5) in all of them. This substitution was reported to be a polymorphism (Chedid *et al*., 1994; Bhatia *et al*., 1995).

In order to find out whether p21/*Waf1* deletions were also present in the genomic DNA of those samples, Southern blot analysis was performed. As shown in Figure 4, no apparent gene deletion, rearrangement or amplification was present, indicating that abnormal RNA splicing may be involved in the deletion of the p21/*Waf1* gene.

Given that all the deletions are located within the second exon of the p21/*Waf1* gene (Figure 5), we next amplified by PCR the second exon from tumour genomic DNA and examined the intron–exon boundary and the coding region to see if there are any mutations which may lead to aberrant RNA splicing. The PCR products were cloned into a TA vector and a minimum of five clones from each sample were sequenced. As shown in Figure 5, the entire second exon was skipped in mutant A and partial second exon was spliced out, probably by using potential cryptic splice sites: 5′-CA....AG-3′ in mutant B and 5′-AC....TG-3′ in mutant C, respectively, although no mutations were found at splice sites surrounding the second exon or within the exon. However, one point mutation (C to G) was found in the second intron 16 bp from 5′ splice site. This mutation was present in samples with

**Figure 1** Analysis of RT–PCR products from thyroid tumour specimens. cDNA fragments were generated by RT–PCR using the primers covering the coding region of the p21/*Waf1* gene. The PCR products were size fractionated on 1.8% agarose gel. The length of the products is indicated on the right. The DNA marker is 1 kb ladder from Gibco BRL (lane 1). Lane 2, PCR product from the wild-type p21/*Waf1* gene. The smaller sized fragments (100–220 bp) amplified in thyroid carcinoma samples are shown in lanes 3, 4 and 5 respectively.

**Figure 2** DNA sequence autoradiographs showing p21/*Waf1* deletion in thyroid carcinomas. Three different deletions were identified: mutants **a**, **b** and **c**. The deletion point is marked by an arrow. Sequencing reactions were performed using single cDNA clone as a template.

**Figure 3** Schematic representation of p21/*Waf1* cDNA deletion in thyroid carcinoma. The wild-type (WT) p21/*Waf1* cDNA is shown at the top, and three different kinds of deletion mutants are shown below. The hatched bar and line represent the coding and untranslated regions respectively. The dark bars represent the flanking primers used to amplify the full-length p21*Waf1* cDNA.

**Figure 4** Southern blot analysis of genomic DNA from thyroid carcinoma specimens. Genomic DNA (10 $\mu$g) digested with *Eco*RI was fractionated on 1.0% agarose gel and transferred to a nylon membrane. Hybridisation was carried out with a full-length p21/*Waf1* cDNA probe (**a**). The same blot was rehybridised with a human thyrotropin receptor cDNA probe to monitor the sample loading (**b**). Lanes 1–5 are tumour specimens with p21/*Waf1* mutations. Lanes 6 and 7 are normal control.

mutant A and C deletions as well as in one of three mutant B deletion samples. It was not present in three normal samples, but was found in one thyroid cancer sample without p21/*Waf1* deletion. Therefore, the mutation may be a polymorphism and whether it contributes to the aberrant RNA splicing remains to be determined. It is worth noting that the 5′ splice point of mutant C happens to be in the third nucleotide of the polymorphic codon 31 (AGC/A), and the third nucleotide is adenine instead of cytosine (Figure 5). Adenine would thus serve as a 5′ splice donor site of mutant C. However, we do not know whether this is a chance event or plays any role in abnormal RNA splicing. Two separate PCRs from original tumour specimens were performed to rule out the possibility of enzymatic errors in PCR amplification with *Taq* polymerase. The results were identical in the separate experiments.

Ten per cent (5/49) of thyroid carcinomas (12.5% of papillary thyroid carcinomas) in this series harboured p21/*Waf1* deletions. Among the five p21/*Waf1* deletion samples, none of them was found to have either a p53 (Zou *et al.*, 1993) or Rb mutation (Zou *et al.*, 1994). Thus, p21/*Waf1* appears to be deleted only in those samples without p53 or

**Figure 5** Partial nucleotide sequences of three p21/*Waf1* mutants and intron–exon boundary of p21/*Waf1* second exon. (**a**) nucleotide sequences showing a point mutation in the second intron (gel 1) and polymorphism at codon 31 (gel 3). The mutation and polymorphism are indicated by an asterisk. The 5′ splice site of mutant C is marked by a horizontal line and happens to be in the third nucleotide of codon 31 (gel 3). Gels 2 and 4 are normal controls used for comparison with gels 1 and 3. (**b**) schematic diagram of splicing patterns. The open bars indicate exons which are missing in the cDNA. The upper-case and lower-case letters represent exon and intron sequences respectively. The splice points are indicated by a vertical line and the primers used to amplify the second exon are underlined. The premature stop codon resulting from abnormal splicing in mutant B is also underlined. Mutated or polymorphic nucleotides are shown below their wild-type sequence.

**Table I** Genetic defects of p53, Rb and p21 in thyroid tumours

| *Tumour* | *Rb gene mutation*[a] *(positive/tested)* | *p53 gene mutation*[b] *(positive/tested)* | *p21 gene deletion (positive/tested)* | *Both p53 and Rb gene mutation (positive/tested)* | *Both p21 and p53 or Rb gene mutation (positive/tested)* |
|---|---|---|---|---|---|
| Adenoma | 0/8 | 0/8 | 0/8 | | |
| Follicular carcinoma | 2/4 | 1/4 | 0/4 | 1/4 | 0/4 |
| Papillary carcinoma | 22/40 | 10/40 | 5/40 | 4/40 | 0/40 |
| Anaplastic carcinoma | 3/5 | 1/5 | 0/5 | 1/5 | 0/5 |

[a]Zou *et al.* (1994). [b]Zou *et al.* (1993a).

Rb mutations. The information regarding genetic defects of p53, Rb and p21 in this series of tumours is summarised in Table I.

### Discussion

We have examined the entire coding region of p21/*Waf1* cDNA for deletions and/or point mutations by RT–PCR and SSCP in 57 thyroid tumour specimens (eight benign adenomas and 49 carcinomas, including 40 papillary carcinomas). Subtle p21/*Waf1* deletions have been found in five papillary carcinoma specimens. The deletions are involved in the most evolutionarily conserved region (nt 63–180 and nt 390–492) (Huppi *et al.*, 1994), and the truncated p21/*Waf1* proteins are probably not functional. El-Deiry *et al.* (1993) previously reported that introduction into human tumour cells of a mutant p21/*Waf1* with a stop codon at nt 222 did not result in significant growth suppression, thus experimentally confirming that the region beyond nt 222 is required for p21/*Waf1* function. More defined p21 binding domains involved in the inhibition of Cdk2 and PCNA have recently been identified (Chen *et al.*, 1995; Goubin and Ducommun, 1995). The Cdk2 binding domain was located in the N-terminal part of the protein, between residues 45 and 60 (nt 135–180). A proliferating cell nuclear antigen (PCNA) binding region was mapped to the C-terminus of the protein between residues 142 and 163 (nt 426–489). Cdk2 is required in the $G_1$–S transition and is found in association with $G_1$ cyclins (D1, D3 and E) implicated in the control of passage through the restriction point of cell cycle (Xiong *et al.*, 1992; Koff *et al.*, 1992; Dulic *et al.*, 1992; Pagano *et al.*, 1993). PCNA is the auxilliary protein of DNA polymerase delta (Bravo *et al.*, 1987) and is thought to be involved in DNA replication and repair processes (Prelich *et al.*, 1987; Shiviji *et al.*, 1992). p21/*Waf1* interacts with and inhibits both Cdk2 and PCNA, thus controlling cell cycle progression and DNA replication (Waga *et al.*, 1994). All three kinds of mutants have lost both Cdk2 and PCNA binding domains. They, therefore, cannot express their inhibitory activity on either Cdk2 or PCNA.

Although normal-sized products were present in the five p21/*Waf1* mutant samples, the wild-type allele might be derived from contaminating non-cancerous cells, such as infiltrating inflammatory cells and stromal cells. Another possibility is that both wild-type and mutant p21/*Waf1* alleles are present in some of the thyroid cancer specimens. This has been supported by a recent report (Bhatia *et al.*, 1995) that a heterozygous point mutation at codon 63 (Phe to Leu) of p21/*Waf1* was found in a Burkitt's lymphoma cell line. Both wild-type and mutant p21/*Waf1* mRNA were expressed in the tumour cell line. Transfection experiments showed that the mutant was less efficient in suppressing clonogenicity than the wild-type. It is, therefore, possible that one wild-type allele of p21/*Waf1* may not function sufficiently to cause $G_1$ arrest, thus resulting in uncontrolled cell cycle progression and tumour formation.

Given that gene deletion and rearrangement are not found in the tumour genomic DNA, abnormal RNA splicing is probably the cause of p21/*Waf1* deletion. The entire second exon is skipped in mutant A. The common causes for exon skipping include: (1) mutations in the conserved sequence at the 5′ donor site or 3′ acceptor site (Green, 1986; Zielenski *et al.*, 1995). Most reported cases of exon skipping are due to a single base substitution that changes the universal AG dinucleotide at the 3′ splice site of the intron (Green, 1986; (2) mutations in the exon (Dietz *et al.*, 1993; Matsuo *et al.*, 1991; Wakamatsu *et al.*, 1992). The exonic mutations would either activate a cryptic splice site (Matsuo *et al.*, 1991; Wakamatsu *et al.*, 1992), or interfere with splicing factor binding or RNA secondary structure (Dietz *et al.*, 1993). Although we could not find any mutations in the conserved splicing sequences surrounding the second exon and within the exon, some uncommon events such as mutations in the second intron or beyond the region we have examined may participate in the exon skipping. The potential cryptic 5′ splice site (5′-CA....AG-3′) used in mutant B and both splice sites (5′-AG...TG-3′) in mutant C do not follow the 5′GT....AG-3′ rule. The GT–AG rule describes the splicing junctions of eukaryotic nuclear genes and is highly conserved in most, if not all, mammalian cells (Padgett *et al.*, 1986). Utilisation of unconserved cryptic splice sites in mutants B and C may suggest that a single or multiple splicing factor defect may be involved in the abnormal splice site selection, and a partial second exon deletion.

In the present study, we were unable to find any samples having both p21/*Waf1* and p53 or Rb mutations. It is known that radiation induces thyroid tumorigenesis, and recent data have shown that radiation-induced $G_1$ arrest in thyroid cells is selectively mediated by the p53–p21/*Waf1* pathway (Namba *et al.*, 1995). p-53induced p21/*Waf1* participates in the $G_1$/S checkpoint, and inhibits multiple Cdks, including Cdk4, which stimulates cell division by phosphorylation of Rb and release of E2F transcription factor. Loss of p21/*Waf1* would probably affect both p53 and Rb tumour-suppressor activities. Thus, p21/*Waf1* mutation alone may be sufficient to induce thyroid malignancy.

### References

BHATIA K, FAN S, SPANGLER G, WEINTRAUB M, O'CONNOR PM, JUDDE J-G AND MAGRATH I. (1995). A mutant p21 cyclin-dependent kinase inhibitor isolated from a Burkitt's lymphoma. *Cancer Res.*, **55**, 1431–1435.

BRAVO R, FRANK R, BLUNDELL PA AND MACDONALD-BRAVO H. (1987). Cyclin/PCNA is the auxilliary protein of DNA polymerase-$\delta$. *Nature*, **326**, 515–517.

CHEDID M, MICHELI P, LENGEL C, HUPPI K & GIVOL D. (1994). A single nucleotide substitution at codon 31 (Ser/Arg) defines a polymorphism in a highly conserved region of the p53-inducible gene WAF1/CIP1. *Oncogene*, **9**, 3021–3024.

CHEN J, JACKSON PK, KIRSCHNER MW AND DUTTA A. (1995). Separate domains of p21 involved in the inhibition of Cdk kinase and PCNA. *Nature*, **374**, 386–388.

DIETZ H, VALLE D, FRANCOMANO CA, KENDZIOR RJ, PYERITZ RE AND CUTTING GR. (1993). The skipping of constitutive exons *in vivo* induced by nonsense mutations. *Science*, **259**, 680–683.

DULIC V, LEES E AND REED SR. (1992). Association of human cyclin E with a periodic G1-S phase protein kinase. *Science*, **257**, 1958–1961.

EL-DEIRY WS, TOKINO T, VELCULESCU VE, LEVY DB, PARSONS R, TRENT JM, LIN D, MERCER WE, KINZLER KW AND VOGELSTEIN B. (1993). WAF1, a potential mediator of p53 tumor suppression. *Cell*, **75**, 817–825.

FARID NR, SHI Y AND ZOU MJ. (1994). Molecular basis of thyroid cancer. *Endocrinol. Rev.*, **15**, 202–232.

GOUBIN F AND DUCOMMUN B. (1995). Identification of binding domains on the p21$^{cip1}$ cyclin-dependent kinase inhibitor. *Oncogene*, **10**, 2281–2287.

GREEN MR. (1986). Pre-mRNA splicing. *Annu. Rev. Genet.*, **20**, 671–708.

HARPER JW, ADAMI GR, WEI N, KEYOMARSI K AND ELLEDGE SJ. (1993). The p21 cdk-interacting protein Cip1 is a potent inhibitor of G1 cyclin-dependent kinases. *Cell*, **75**, 805–816.

HERMANEK P AND SOBIN LH. (1987). *TNM Classification of Malignant Tumors*. pp. 33–35, Springer-Verlag: Berlin.

HUNTER T AND PINES J. (1994). Cyclins and cancer II: cyclin D and CDK inhibitors come of age. *Cell*, **79**, 573–582.

HUPPI K, SIWARSKI D, DOSIK J, MICHIELI P, CHEDID M, REED S, MOCK B, GIVOL D AND MUSHINSKI JF. (1994). Molecular cloning, sequencing, chromosomal localization and expression of mouse p21 (Waf1). *Oncogene*, **9,** 3017–3020.

JEN J, HARPER JW, BIGNER SH, BIGNER DD, PAPADOPOULOS N, MARKOWITZ S, WILLSON JKV, KINZLER KW AND VOGELSTEIN B. (1994). Deletion of p16 and p15 genes in brain tumors. *Cancer Res.*, **54,** 6353–6358.

KAMB A, GRUIS NA, WEAVER-FELDHAUS J, LIU Q, HARSHMAN K, TAVTIGIAN SV, STOCKERT E, DAY RS III, JOHNSON BE AND SKOLNICK MH. (1994). A cell cycle regulator potentially involved in the genesis of many tumor types. *Science*, **264,** 436–440.

KOFF A, GIORDANO A, DESIA D, YAMASHITA K, HARPER JW, ELLEDGE SJ, NISHIMOTO T, MORGAN DO, FRANZA R AND ROBERTS JM. (1992). Formation and activation of cyclin E–cdk2 complex during the G1 phase of the human cell cycle. *Science*, **257,** 1689–1694.

MATSUO M, MASUMURA T, NISHIO H, NAKAJIMA T, KITOH Y, TAKUMI T, KOGA J AND NAKAMURA H. (1991). Exon skipping during splicing of dystrophin mRNA precursor due to an intraexon deletion in the dystrophin gene of Duchenne muscular dystrophy. *Kobe. J. Clin. Invest.*, **87,** 2127–2131.

MORI T, MIURA K, AOKI T, NISHIHIRA T, MORI S AND NAKAMURA Y. (1994). Frequent somatic mutation of the MTS1/CDK4I (multiple tumor suppressor/cyclin-dependent kinase 4 inhibitor) gene in esophageal squamous cell carcinoma. *Cancer Res.*, **54,** 3396–3397.

NAMBA H, HARA T, TUKAZAKI T, MIGITA K, ISHIKAWA N, ITO K, NAGATAKI S AND YAMASHITA S. (1995). Radiation-induced G1 arrest is selectively mediated by the p53-WAF1/Cip1 pathway in human thyroid cells. *Cancer Res.*, **55,** 2075–2080.

NORBURY C AND NURSE P. (1992). Animal cell cycles and their control. *Annu. Rev. Biochem.*, **61,** 441–470.

ORITA M, IWAHANA H, KANAZAWA H, HAYASHI K AND SEKIYA T. (1989*a*). Detection of polymorphisms of human DNA by gel electrophoresis as single-strand conformation polymorphisms. *Proc. Natl Acad. Sci. USA*, **86,** 2766–2770.

ORITA M, SUZUKI Y, SEKIYA T AND HAYASHI K. (1989*b*). Rapid and sensitive detection of point mutations and DNA polymorphisms using the polymerase chain reaction. *Genomics*, **5,** 874–879.

PADGETT RA, GRABOWSKI PJ, KONARSKA MM, SEILER S AND SHARP PA. (1986). Splicing of messenger RNA precursors. *Annu. Rev. Biochem.*, **55,** 1119–1150.

PAGANO M, PEPPERKOK J, LUKAS V, BALDIN W, ANORGE J, BARTEK B AND DRAETTA G. (1993). Regulation of the human cell cycle by the cdk2 protein kinase. *J. Cell Biol.*, **121,** 101–111.

PRELICH G, KOSTURA M, MARSHAK DR, MATHEWS MB AND STILLMAN B. (1987). The cell-cycle regulated proliferating cell nuclear antigen is regulated for SV40 DNA replication *in vitro*. *Nature*, **326,** 471–475.

SAMBROOK J, FRITSCH EF AND MANIATIS TE. (1989). *Molecular Cloning: A Laboratory Manual.* Cold Spring Harbor Laboratory Press: Cold Spring Harbor, New York.

SCHMIDT EE, ICHIMURA K, REIFENBERGER G AND COLLINS VP. (1994). CDKN2 (p16/MTS1) gene deletion or CDK4 amplification occurs in the majority of glioblastomas. *Cancer Res.*, **54,** 6321–6324.

SHIOHARA M, EL-DEIRY WS, WADA M, NAKAMAKI T, TAKEUCHI S, YANG R, CHEN D-L, VOGELSTEIN B AND KOEFFLER HP. (1994). Absence of WAF1 mutations in a variety of human malignancies. *Blood*, **84,** 3781–3784.

SHIVJI MKK, KENNY MK AND WOOD RD. (1992). Proliferating cell nuclear antigen is required for DNA excision repair. *Cell*, **69,** 367–374.

WAGA S, HANNON G, BEACH D AND STILLMAN B. (1994). The p21 inhibitor of cyclin-dependent kinases controls DNA replication by interaction with PCNA. *Nature*, **369,** 574–578.

WAKAMATSU N, KOBAYASHI H, MIYATAKE T AND TSUJI S. (1992). A novel exon mutation in the human β-hexosaminidase β subunit gene affects 3′ splice site selection. *J. Biol. Chem.*, **267,** 2406–2413.

XIONG Y, ZHANG H AND BEACH D. (1992). D type cyclins associate with multiple protein kinases and the DNA replication and repair factor PCNA. *Cell*, **71,** 505–514.

XIONG Y, HANNON GJ, ZHANG H, CASSO D, KOBAYASHI R AND BEACH D. (1993). p21 is a universal inhibitor of cyclin kinases. *Nature*, **366,** 701–704.

ZHANG H, XIONG Y AND BEACH D. (1993). Proliferating cell nuclear antigen and p21 are components of multiple cell cycle kinase complexes. *Mol. Biol. Cell.*, **4,** 897–906.

ZIELENSKI J, MARKIEWICZ D, LIN S-P, HUANG F-Y, YANG-FENG TL AND TSUI L-C. (1995). Skipping of exon 12 as a consequence of a point mutation (1898 + 5G→T) in the cystic fibrosis transmembrane conductance regulator gene found in a consanguineous Chinese family. *Clin. Genet.*, **47,** 125–132.

ZOU MJ, SHI Y AND FARID NR. (1993*a*). p53 mutations in all stages of thyroid carcinomas. *J. Clin. Endocrinol. Metab.*, **77,** 1054–1058.

ZOU MJ, SHI Y, AL-SEDAIRY ST AND FARID NR. (1993*b*). High levels of Nm23 gene expression in the advanced stage of thyroid cancer. *Br. J. Cancer*, **68,** 385–388.

ZOU MJ, SHI Y AND FARID NR. (1994). Frequent inactivation of the retinoblastoma gene in human thyroid carcinomas. *Endocrine*, **2,** 193–198.

ZOU MJ, SHI Y, AL-SEDAIRY ST, HUSSAIN SS AND FARID NR. (1995). The expression of the MDM2 gene, a p53 binding protein in thyroid carcinogenesis. *Cancer*, **76,** 314–318.